# Communities of Phytoplankton Viruses across the Transition Zone of the St. Lawrence Estuary

**DOI:** 10.3390/v10120672

**Published:** 2018-11-27

**Authors:** Myriam Labbé, Frédéric Raymond, Alice Lévesque, Mary Thaler, Vani Mohit, Martyne Audet, Jacques Corbeil, Alexander Culley

**Affiliations:** 1 Département de Biochimie, de Microbiologie et de Bio-Informatique, Université Laval, Québec City, QC G1V 0A6, Canada; myriam.labbe.6@ulaval.ca (M.L.); alice.levesque.1@ulaval.ca (A.L.); mary.thaler.1@ulaval.ca (M.T.); vani.mohit@sn.ulaval.ca (V.M.); martyne.audet@gmail.com (M.A.); 2 Centre de Recherche du Centre Hospitalier de l’Université Laval-Québec, Québec City, QC G1V 4G2, Canada; Frederic.Raymond@crchudequebec.ulaval.ca (F.R); jacques.corbeil@genome.ulaval.ca (J.C.)

**Keywords:** aquatic viruses, DNA Polymerase B, *Phycodnaviridae*, *Picornavirales*, RNA-dependent RNA polymerase, St. Lawrence Estuary, viral ecology

## Abstract

The St. Lawrence hydrographic system includes freshwater, brackish, and marine habitats, and is the largest waterway in North America by volume. The food-webs in these habitats are ultimately dependent on phytoplankton. Viral lysis is believed to be responsible for a major part of phytoplankton mortality. To better understand their role, we characterized the diversity and distribution of two viral taxa infecting phytoplankton: the picornaviruses and phycodnaviruses. Our study focused on the estuary transition zone, which is an important nursery for invertebrates and fishes. Both viral taxa were investigated by PCR amplification of conserved molecular markers and next-generation sequencing at six sites, ranging from freshwater to marine. Our results revealed few shared viral phylotypes between saltwater and freshwater sites. Salinity appeared to be the primary determinant of viral community composition. Moreover, our analysis indicated that the viruses identified in this region of the St. Lawrence diverge from classified viruses and homologous published environmental virotypes. These results suggest that DNA and RNA viruses infecting phytoplankton are likely active in the estuary transition zone, and that this region harbors its own unique viral assemblages.

## 1. Introduction

The St. Lawrence drainage basin is one of the largest freshwater hydrographic systems in the world. This aquatic system is comprised of a network of freshwater, brackish, and marine habitats that supports a rich diversity of organisms [1]. Where freshwater from the St. Lawrence drainage basin encounters seawater from the Gulf of St. Lawrence, there is an estuarine transition zone (ETZ). The ETZ experiences estuarine recirculation, semi-diurnal stratification, and tidal mixing, and is characterized by high turbidity and sharp gradients, in particular of salinity, temperature, and photosynthetically active radiation [1]. These factors contribute to high levels of zooplankton biomass [2], which in turn support nurseries of fish species of commercial interest [3]. The composition of phytoplankton in this ecotone reflects the transition from freshwater to seawater. Lapierre and Frenette [4] found a decrease in cyanobacterial abundance corresponding with an increase in marine diatom taxa as salinity increased, whereas Lovejoy et al. [2] found that photosynthetic picoplankton declined in abundance moving downstream.

The relative importance of in situ primary production versus the advection of organic material has been debated. In some parts of the estuary, the depth of mixing is several meters below the euphotic depth, limiting the light available for photosynthesis, while seasonal plumes of freshwater from the St. Lawrence, Saguenay, and other rivers can flush phytoplankton downstream [5]. While Vincent et al. [6] calculated that 20–30% of the phytoplankton at the turbidity maximum is advected from upstream, they found a contribution of in situ photosynthesis that was as high as or higher than allochthonous production, in contrast to other estuarine systems where the historical viewpoint has been that bacteria are the dominant source of total production in the ETZ [7]. Phytoplankton comprise a relatively low percentage of the total ETZ particulate organic carbon (roughly 10%), but it appears that they are selectively grazed by primary consumers [8]. Given the importance of phytoplankton in this system, an understanding of the factors that influence the dynamics and interactions of this group is vital to understanding the ecology of the ETZ.

Viruses have an important role controlling phytoplankton populations [9], and along with grazing, are one of the two major sources of phytoplankton mortality in aquatic environments. Several studies have demonstrated that a diverse variety of grazers is active in the ETZ [10,11], but the role of viral lysis is less well-understood. The literature on viruses in fluvial systems contains major gaps, particularly on knowledge of eukaryote viruses, their diversity and effect on host diversity, and horizontal transport of virus particles [12].

*Phycodnaviridae* is a family of large, double-stranded DNA viruses. Viruses in this group that have been cultured infect most major phytoplankton phyla, including chlorophytes, haptophytes, dinoflagellates, and the brown algae macrophyte *Ectocarpus siliculosus* [13]. A putative phycodnavirus infecting the cryptophyte *Teleaulax amphioxeia* has also been described, though sequence data are lacking [14].

In contrast, though RNA viruses have been known to infect metazoans and heterotrophic bacteria, evidence of RNA-virus infections among protists is fairly new [15]. The description of positive-sense ssRNA viruses infecting toxic bloom-forming dinoflagellate *Heterocapsa circularisquama* [16], as well as an RNA virus infecting a diatom, have shed new light on this aspect of marine virology [15,17]. Most of the RNA viruses infecting phytoplankton to date have been classified in the order of *Picornavirales* [18]. Not only have phytoplankton-infecting RNA viruses been discovered, but it has been observed that a large part of the marine viral community is composed of RNA viruses, making them as abundant as viruses with DNA genomes [19]. While more marine viruses and their hosts are being characterized, the sequence databases of marine viruses remain impoverished, making it difficult to identify sequences through similarity or phylogeny [17].

We characterized the phycodnavirus and picornavirus communities along a salinity gradient in the St. Lawrence Estuary using group-specific primers. In order to assess the diversity of their putative hosts—the eukaryote community—we also sequenced both the transcribed 18S rRNA, to characterize eukaryotes that are actively growing and dividing (“active community”), and the 18S rRNA gene, to characterize all eukaryotes present in the sample (“total community”). Given that known viruses in these taxa are lytic, strain specific, and likely have a low residence time, we expected very little overlap in taxa among the distinctive aquatic environments sampled along the transition-zone continuum.

## 2. Materials and Methods

### 2.1. Sampling Locations

**Figure 1:**
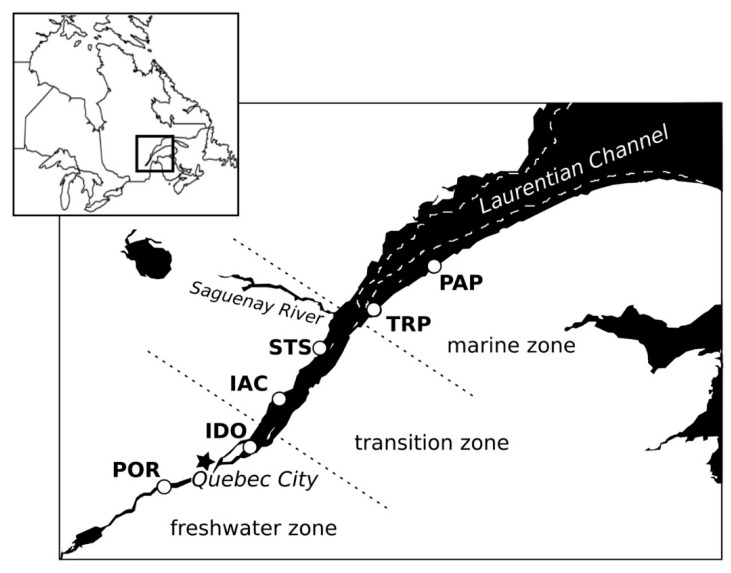
Location of sampling sites in the St. Lawrence estuary. The Laurentian Channel is delimited by a broken line.

Measuring some 1200 km in length, the St. Lawrence River can be considered as a partly-mixed estuarine system, divided into three salinity-defined zones: the freshwater zone, originating in the North American Great Lakes; the marine zone, which terminates downstream in the Gulf of St. Lawrence; and a transition zone between these two. In the transition zone, higher-density saltwater flows beneath freshwater, and sediment re-suspension by tidal currents results in a zone of maximum turbidity, with high nutrient concentrations [11]. The transition zone presents a high diversity of both heterotrophic and autotrophic micro-organisms. Our sampling sites are roughly equidistant (55–90 km) along a 327 km line from the marine zone (Pointe-au-Père (PAP) and Trois-Pistoles (TRP)), through the transition zone (St-Siméon (STS) and Isle-aux-Coudres (IAC)), and ending in the freshwater zone (Île d’Orléans (IDO) and Portneuf (POR)) (Figure 1). All of our sites are within 280 km of Québec City, where samples were analyzed at Université Laval.

### 2.2. Sample Collection and Filtration

Samples were collected from the six sites over 15–18 July 2014, between 4:15 a.m. and 2:15 p.m. at high tide to minimize suspended sediments. Duplicate samples of surface water were collected with a stainless-steel bucket, transferred into 1 L Nalgene bottles which had been rinsed with sample water, and filtered on the same day. Physical properties of the water column were measured using a SEACAT SBE 19-03 (SeaBird electronics). For salinity, two measurements were taken at each site between 0.3 m and 2.2 m, and the mean of the values was used. Tubing for filtration was cleaned with 2% Contrad 70 detergent (DeCon Labs, Philadelphia, PA, USA) and rinsed with MilliQ water and sample water. Water was filtered with a peristaltic pump onto 25 mm Whatman Anotop 0.02 µm aluminum oxide filters, either as whole-water samples, or following pre-filtration on a 0.22 µm Sterivex filter cartridge (Millipore). Filtration was stopped when no more water could pass through the filter. The volume filtered at each site varied from 59 to 425 mL, and was lower at turbid stations. Filters were stored at −80 °C.

### 2.3. Nucleic-Acid Extraction

Nucleic acids were extracted from Anotop filters with an Epicentre MasterPure Complete DNA & RNA Purification Kit (Epicentre Biotechnologies, Madison, WI, USA) using “back flushing”, as described by Mueller et al. [20]. Briefly, 1 mL Tissue and Cell Lysis solution from the kit with 100 µg mL^−1^ proteinase K was injected into the Anotop filter outlet using a sterile 3 mL syringe. A second syringe was connected to the inlet of the filter. This assembly was incubated for 15 minutes at 65 °C. The solution was then aspirated into the syringe through the filter inlet, transferred into a 1.5 mL microtube, and kept on ice for 3–5 minutes. This volume was then divided into two 500 µL fractions, from which nucleic acids were extracted following the manufacturer’s protocol with minor adjustments for the different volumes, where we used 275 µL of the Protein Precipitation Reagent, and 750 µL of isopropanol. When pelleting the nucleic acids, the entire volume was pelleted in one tube, with two sequential centrifugations in order to obtain a higher concentration of nucleic acids in a single tube. After the washing step, residual ethanol was removed by evaporation for about 17 h at room temperature. The pellet of nucleic acids was re-suspended in 50 µL of sterile water (Sigma Aldrich, USA). Since preliminary tests indicated PCR inhibition, possibly by humic substances in the sample water, the samples were additionally cleaned using the PowerClean Pro RNA Clean-up Kit and PowerClean Pro DNA Clean-up Kit (MoBio) according to the manufacturer’s protocol.

RNA was extracted only from samples collected with pre-filtration, not from whole-water samples. 20 µL of the extracted nucleic acids was transferred into a new tube and treated with a TURBO DNA-free Kit. Extracted RNA was converted to cDNA using a SuperScript III First-Strand Synthesis System (Invitrogen) with 100 ng µL^−1^ random hexamer primers, and treated with RNase H according to the manufacturer’s protocol.

### 2.4. High-Throughput Sequencing of Viral Nucleic Acids

The active site locus of the RNA-dependent RNA-polymerase (RdRp) gene was targeted using *Picornavirales*-specific primers (Table 1), and the Expand High Fidelity PLUS PCR System. 10 µL of reverse-transcription product was added to a total reaction volume of 50 µL, with 1 µM each of the forward and reverse primers. Thermal cycling consisted of an initial 2 minutes at 94 °C, followed by 40 cycles of 30 s at 94 °C, 30 s at a primer-specific hybridization temperature (Table 1), and 1 minute at 72 °C, finishing with a final elongation step of 7 minutes at 72 °C. PCR products were visualized by migrating on a 1% agarose with a SYBRSafe stain (Invitrogen).

The DNA-dependent DNA polymerase gene (DNA Pol), also known as “Polymerase B”, was targeted with specific primers (Table 1) for both pre-filtered and whole-water samples, following the same PCR protocol and visualization given above. Primers targeting the Major Capsid Protein [24] were also tested on the samples, but yielded no visible band.

Bands in the 400–600 bp range for RdRp primers and 700–850 bp for DNA Pol primers were excised using a blue light filter to prevent degradation by UV, and purified using the Qiagen MinElute Gel Purification Kit (Qiagen). Products were re-amplified using the same PCR procedure as above, but with only 25 cycles, and re-purified before sequencing.

PCR products were prepared for high-throughput sequencing using a Nextera DNA Library Preparation Kit (Illumina) which fragmented amplicons and added Illumina sequencing adaptors. Amplicons were pair-end sequenced on the HiSeq Illumina system at the Centre de Recherche du Centre Hospitalier de l’Université Laval, Québec, Canada. The raw reads have been made available in the NCBI Sequence Read Archive (SRA; http://www.ncbi.nlm.nih.gov/sra) under the accession number PRJNA382556.

### 2.5. 18S rRNA and 18S rDNA High-Throughput Sequencing

The V4 region of the protist eukaryotic 18S rRNA and 18S rRNA gene (rDNA) was amplified through an initial PCR using the primers and amplification protocol described in Comeau et al. [25], except that Illumina adaptors were appended to the ends of the primers. Three different dilutions from whole-water samples were used for the amplification. Amplicon size was verified by running the PCR products on 1% agarose gels. PCR products from the same sample were pooled together and purified using Axygen magnetic beads (Corning Life Sciences, NY, USA). The purified product was eluted in elution buffer (EB buffer; 10 mM Tris-HCl, pH 8, Qiagen, Germany) and quantified with a nanodrop ND-1000. The purified amplification products were diluted 10–50× based on the quantification results. A second PCR on the diluted products was performed with index primers from Illumina. The PCR protocol was as follows: initial denaturation at 98 °C for 30 s, 13 cycles of denaturation at 98 °C for 10 s, annealing at 55 °C for 30 s, elongation at 72 °C for 30 s, and a final elongation at 72 ° C for 4 minutes 30 s. After a second purification step, the amplified products were quantified spectrophotometrically and pooled equimolarly. The pool was paired-end sequenced on the MiSeq Illumina system at the Institut de Biologie Intégrative et des Systèmes, Université Laval Plate-forme d’Analyses Génomiques, Québec, Canada. The raw reads have been made available in the National Centre for Biotechnology Information (NCBI) Sequence Read Archive (SRA; http://www.ncbi.nlm.nih.gov/sra) under the accession number PRJNA382556.

### 2.6. Analysis of Reads

For viruses, reads were trimmed to remove adaptors and low-quality reads using Trimmomatic [26], and reads from all samples were assembled together using Ray Meta [27]. Each contig was treated as an Operational Taxonomic Unit (OTU). Trimmed, unassembled reads were classified using BLAST (basic local alignment search tool) in DIAMOND (double index alignment of next-generation sequencing data) [28], and cellular reads were eliminated at this step. Filtered reads were then pseudo-aligned to a reference dataset of contigs using kallisto [29] and assigned to an OTU/contig, as described by Steward et al. [19]. Finally, contigs were screened for length. Inteins in viral DNA Pol are known to result in unusually long amplicons. However, the inteins described so far for phycodnaviruses all possess a conserved flanking region with the sequence YGD-TDS found in both mimiviruses and phycodnaviruses [30,31,32]. Two contigs >1000 bp were removed from analysis because they did not have this conserved sequence, and were suspected to result from spurious amplification.

For eukaryotes, raw reads from high-throughput sequencing were processed using the UPARSE pipeline [33] implemented in the USEARCH v9.2.64 software suite [34], including the quality control process to remove low-quality reads and singletons, i.e. reads occurring only once in the whole dataset. OTUs were clustered at 98% sequence identity and classified taxonomically with the mothur taxonomy assigner [35] implemented on the QIIME (Quantitative Insights Into Microbial Ecology) 1.9.1 platform [36] against a custom-curated eukaryotic reference database [37]. OTUs annotated as Fungi, Metazoa, or Streptophyta were eliminated from the dataset.

### 2.7. Statistical Analysis

For all statistical analyses, reads originating from different primers were grouped together for DNA and RNA viruses respectively, as were pre-filtered versus whole-water samples; i.e., no attempt was made to consider the effects of the primer or fractionation.

Before diversity analyses, reads were sub-sampled (rarefied) to 12,000 per sample site for DNA viruses, and 2000 per sample site for RNA viruses. Alpha diversity indices were calculated in PAST (Palaeontological Statistics) v3.15 [38]. Three distinct clustering methods were used to analyze beta diversity: a Jaccard index on raw reads, as implemented in COMMET (Compare Multiple Metagenomes) [39]; a Morisita-Horn index on contigs, classified in DIAMOND; and phylogeny-based distances from unweighted UniFrac [40]. The unweighted UniFrac distances were calculated using mothur, based on a sequence alignment constructed using MAFFT (Multiple Alignment using Fast Fourier Transform) [41] and a phylogenetic tree constructed with Fasttree v2.1.9 [42]. The unweighted pair-group method with arithmetic mean (UPGMA) was used to cluster sites based on UniFrac distances. The robustness of the clusters was assessed via comparison with 1000 jackknifed replicates. A similarity percentage (SIMPER) analysis was performed in PAST to determine which contigs contributed most to the difference between freshwater and brackish/marine communities.

An unweighted UniFrac was performed on eukaryotic OTUs as described above for viruses, except that the samples were rarefied to 22,000 reads, and data from duplicate samples were pooled before performing statistical analyses on the dataset. Preliminary analyses showed similar clustering for RNA and DNA reads, so these were pooled. A SIMPER analysis was performed to determine which eukaryotic taxa contributed the most to the average Bray-Curtis dissimilarity between the clusters formed a priori by UniFrac clustering.

To evaluate the influence of environmental variables on virus distribution, we performed a distance-based redundancy analysis (db-RDA, [43]) using a weighted UniFrac distance matrix, as recommended by Shankar et al. [44] and implemented by the *capscale* function of the R package vegan [45]. Preliminary analysis used the 12 eukaryote taxa identified with SIMPER as environmental variables, and these were used to choose an informative subset of high-level eukaryote taxa which were entered into the model as a proportion of eukaryote OTUs, along with salinity. A few eukaryotic taxa were removed during analysis because they had zero eigenvalues resulting from collinearity with other variables. These were Geminigeraceae (Cryptophyta), Mamiellophyceae (Chlorophyta), and Picozoa, and, for RNA viruses only, Dinoflagellates.

### 2.8. Phylogenetic Analysis

Reference sequences were obtained for the DNA Pol and RdRp genes from GenBank. For DNA Pol, environmental sequences similar to our contigs were also retrieved from the IMG/VR database [46] using BLAST. 66 and 16 sequences for DNA and RNA, respectively, were translated to amino acids and aligned using MAFFT. Positions with very long inteins (>300 bp) were removed from the analysis. DNA and RNA alignments had 1933 and 131 amino acids respectively, including gaps. Note that viral DNA Pol are typically between 900–1300 amino acids long [47], while picornavirus RdRp are 380 amino acids [48]. Reference trees were constructed with 100 bootstraps using RAxML v.8.2.0 [49] and the PROTGAMMAAUTO model, and rooted using *Aeromonas* virus Aeh1 (NP_943895.1) and *Bacillus* virus SPO1 (AAA03732.1) as out-groups for DNA Pol, and the Wheat streak mosaic virus (NC_001886) as an out-group for RdRp. For both communities, the 20 contigs which contributed most to the difference between freshwater and brackish/seawater sites in SIMPER were included in the analysis. For DNA Pol, contigs were mapped onto the reference tree using the Evolutionary Placement Algorithm (EPA) of RAxML [50]. An additional 6 short sequences obtained from Short et al.’s study of Lake Ontario [23] were also mapped onto the DNA Pol tree. For RdRp, because contig and reference sequences were of comparable length, contigs could be included in the maximum likelihood tree without adding an EPA step.

## 3. Results

### 3.1. Environmental Parameters

POR and IDO are freshwater sites with salinities ranging from 0.1 to 0.2, while the remaining sites (IAC, STS, TP, and PAP) were all influenced by saltwater, with salinities from 23.2 to 29.5 (Table 2). While turbidity was not measured, visual observations indicated that the water was very turbid at IDO, less turbid at POR and IAC, and not turbid at STS, TRP, or PAP.

### 3.2. Analysis of Eukaryotic Communities

**Figure 2:**
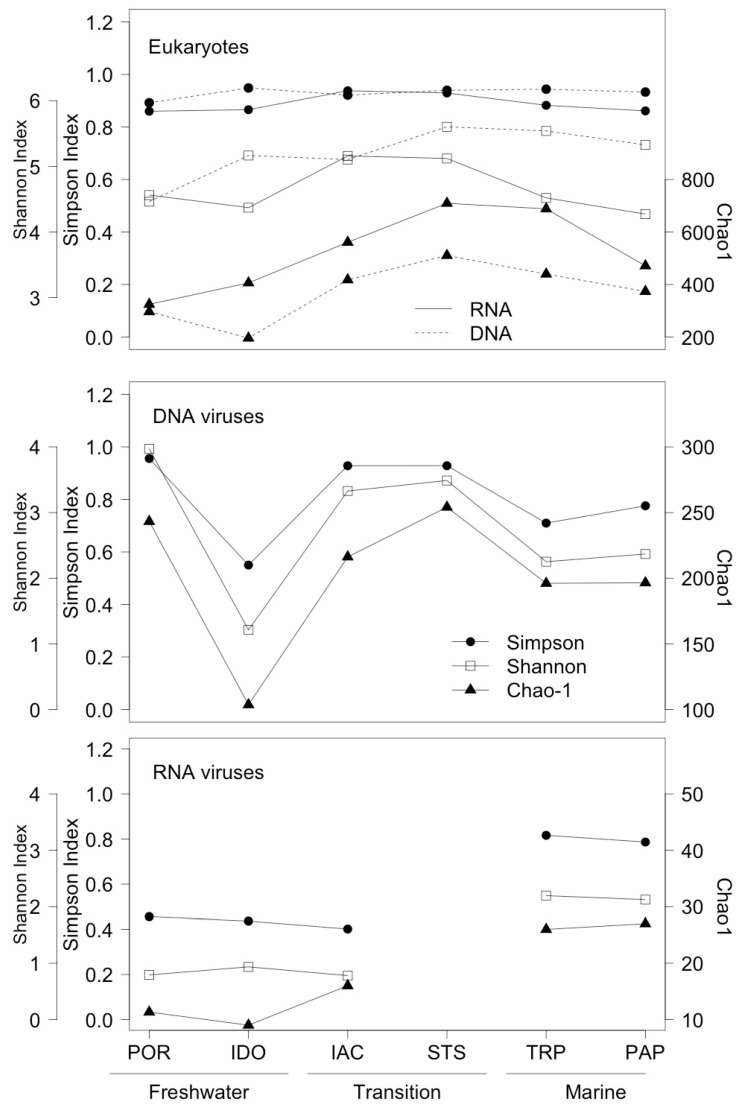
Alpha diversity (Simpson, Shannon, and Chao1 indices) for Eukaryotes (using the 18S rRNA and 18S rDNA gene), DNA viruses, and RNA viruses. Note the changing axes for the Shannon Index and Chao1 Index.

**Figure 3:**
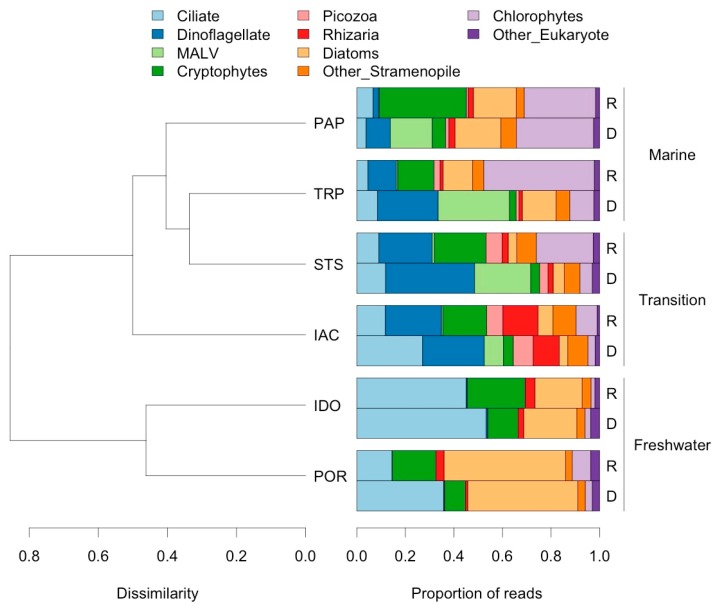
Samples clustered using unweighted UniFrac distances on eukaryote community, and proportion of reads belonging to major taxonomic groups based on 18S rRNA (“R”) and the 18S rDNA gene (“D”).

For eukaryote diversity, the Simpson Index was close to invariant along the salinity gradient, but the two other indices of alpha diversity were highest at the transition site STS for RNA and DNA (Figure 2). The proportions of major taxonomic groups were mostly consistent between “active” and “total” communities for eukaryotes, although some groups, such as cryptophytes and chlorophytes, had a higher relative abundance in the active community, and others, such as Marine Alveolates (MALV) and ciliates, had a higher relative abundance in the total community (Figure 3).

Unweighted UniFrac analysis highlighted the distinctness of freshwater eukaryotic assemblages (Figure 3), while SIMPER analysis identified the taxa responsible for this difference to the genus level (Table 3). Taxa differed slightly in relative importance between the active and total communities. Freshwater sites were characterized by higher proportions of ciliates, diatoms, and cryptophytes, such as *Cryptomonas*. Marine sites were characterized by chlorophytes of the Mamiellophyceae, dinoflagellates, cryptophytes of the Geminigeraceae, and, in the total community only, Marine Alveolates. Transition sites IAC and STS did not cluster together with UniFrac, with STS branching closer to the marine sites. The transition sites shared a higher relative abundance of Picozoa, which were not found in freshwater or marine sites, and IAC had a number of unique features, including a higher proportion of Rhizaria (nearly all cercozoans). Additionally, although overall diatom abundance at IAC was low, relative abundance of the genus *Skeletonema* (4.6% of reads in RNA) was more suggestive of levels at freshwater sites (12.5% and 35%) than marine sites (<1%). SIMPER identified *Skeletonema* as one of the most important taxa contributing to the difference between freshwater and marine sites (Table 3).

### 3.3. Analysis of DNA Pol and RdRP Sequences

Both primer sets specific to RNA viruses amplified cDNA from all sites except STS. This station has been omitted from analysis of RNA viruses. The DNA virus-specific primer set, AVS, amplified DNA from all sites except IDO, and the primer set ChlVd amplified DNA from all sites except TRP. Whole-water samples, which were used only to extract DNA, not RNA, yielded amplicons only for marine sites and the brackish site STS, while pre-filtered samples yielded DNA amplicons at all sites. While preliminary analysis indicated that different primer sets amplify different virus populations (Supplementary Figure S1), these methodological inconsistencies led us to decide not to analyze the effects of different primers or filtration techniques. Once these were pooled, the number of reads per sample site varied by three orders of magnitude for both DNA and RNA viruses (Table 2). To perform meaningful diversity analyses, reads were therefore subsampled to 12,000 for DNA viruses and 2000 for RNA viruses.

Taxonomic richness, defined as the number of different contigs, tended to be higher for DNA viruses than for RNA viruses, most markedly in the freshwater and transition zones (Figure 2). This was not solely due to greater sampling effort, as is demonstrated by the raw data from IAC and IDO, for which a higher number of sequence reads were retrieved for RNA than for DNA; yet, the number of different contigs for RNA was 6–7 times lower (Table 2). The trend in diversity also differed between DNA and RNA viruses. DNA viruses had dramatically lower diversity at IDO compared to other stations, but generally higher diversity at POR (freshwater) and the transition stations, compared to the marine stations. A reverse trend held for RNA viruses, for which alpha diversity was higher at marine than freshwater or transition sites.

**Figure 4:**
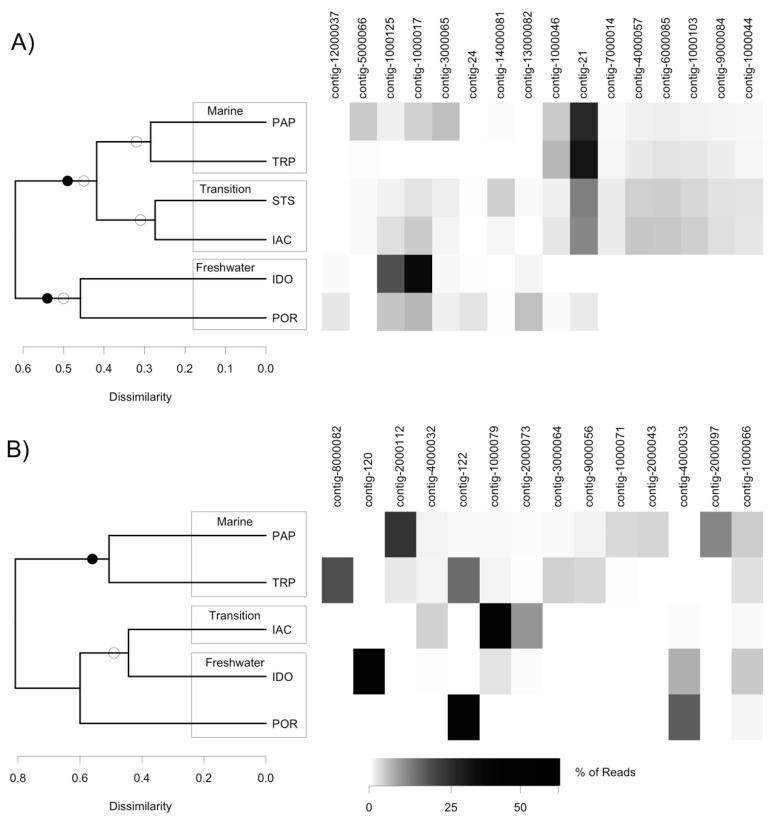
Samples clustered using unweighted UniFrac distances on virus community, and relative abundance of contigs in sampling sites (rarefied to 12,000 reads per sample for DNA viruses and 2000 reads for RNA viruses); (**A**) DNA virus community; (**B**) RNA virus community. Open circles indicate nodes which also appear in clustering using a Morisita-Horn index based on OTUs. Closed circles indicate nodes which also appear in a COMMET (Compare Multiple Metagenomes) analysis (Jaccard index) of raw reads. For COMMET analysis, reads were rarefied to 35,500 for DNA viruses and 7900 for RNA viruses. Note that no RNA viruses were detected at the St-Siméon site (STS).

**Figure 5:**
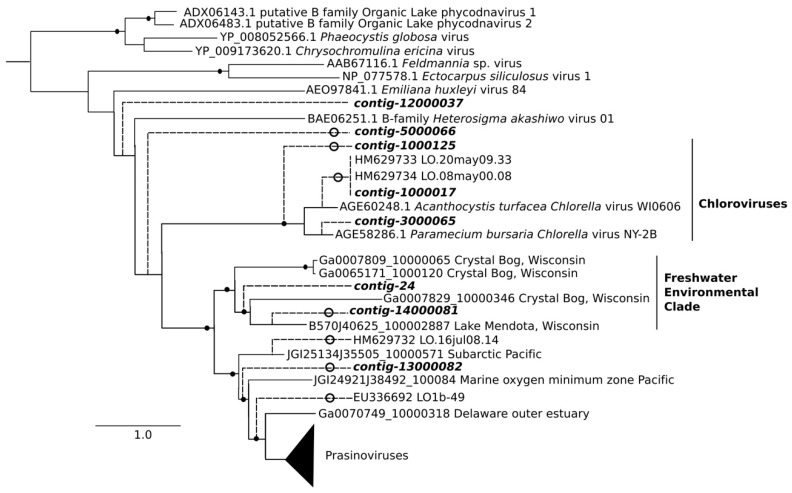
Evolutionary Placement Algorithm (EPA) of the viral DNA Pol contigs which contributed most to the dissimilarity between freshwater and brackish/saltwater samples in the Similarity Percentage (SIMPER) analysis. Closed circles show nodes of reference tree with bootstrap values >50 (out of 100). Open circles show contigs placed by EPA with Likelihood Weight >0.5. Scale bar shows number of substitutions. Branch lengths to contigs (broken lines) are arbitrary. Out-group (not shown) is *Aeromonas* virus Aeh1. The prasinovirus clade, collapsed here for clarity, is shown in full in Figure 6.

The DNA virus communities separated according to salinity-defined zones for nearly all beta-diversity methods, although COMMET analysis was unable to separate out transition- and marine-zone sampling sites (Figure 4A). STS and IAC DNA virus communities can be seen clustering together in both types of analysis, despite the fact that the eukaryotic communities at these sites did not. Although phylogenetic analysis suggested that contig-14000081 came from a putative freshwater clade, it was found exclusively at the two transition sites (Figure 4 and Figure 5).

Compared to the DNA virus community, the RNA virus community had a lower level of shared contigs between sites in the same salinity range (Figure 4). Overall, less than 5% of contigs for DNA viruses were unique to any given site, whereas this proportion was 18–36% for RNA viruses (Table 2). Unweighted UniFrac separated the marine zone samples as a distinct community, while COMMET analysis recovered all three zones as distinct clusters (data not shown). Both Morisita-Horn and unweighted UniFrac recovered a cluster containing IAC and IDO—two sites with strongly different salinity. An extreme dominance by contig-122 in POR seems to have prevented the two freshwater sites from clustering together (Figure 4B).

**Figure 6:**
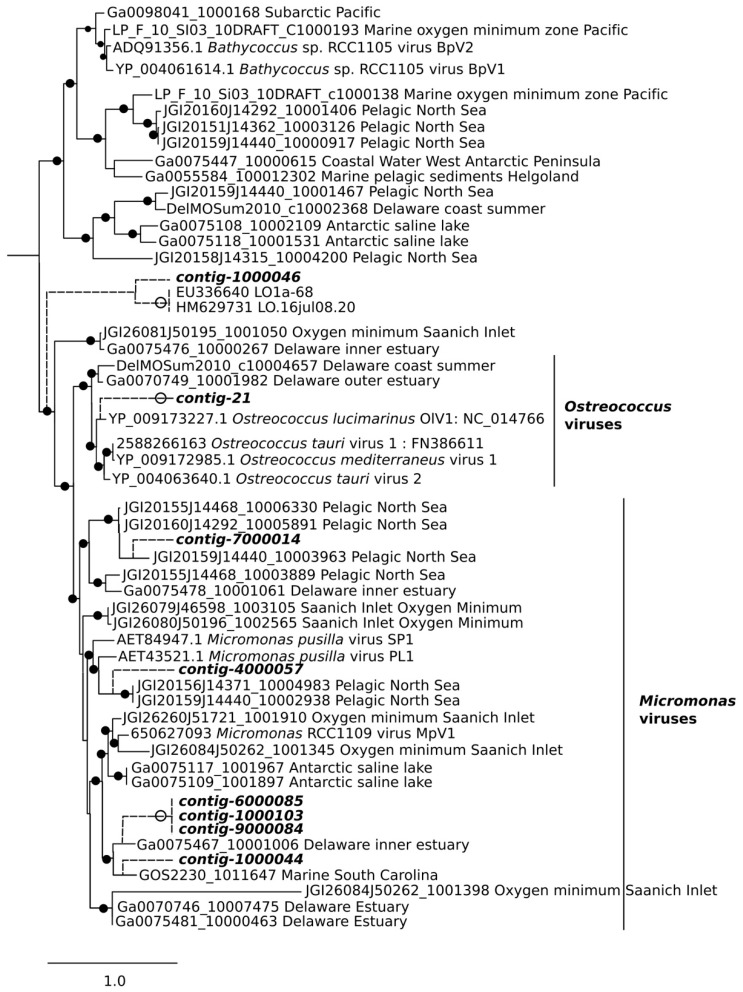
Evolutionary Placement Algorithm (EPA) of the prasinovirus DNA Pol contigs, which contributed most to the dissimilarity between freshwater and brackish/saltwater samples in Similarity Percentage (SIMPER) analysis. Closed circles show nodes of the reference tree with bootstrap values > 50 (out of 100). Open circles show contigs placed by EPA with Likelihood Weight > 0.5. Scale bar shows number of substitutions. Branch lengths to contigs (broken lines) are arbitrary.

Only 46 environmental sequences similar to our DNA virus contigs were retrieved from the IMG/VR database using BLAST. A clade containing only environmental sequences and prasinoviruses was retrieved with high bootstrap support. All of the contigs which EPA placed in this prasinovirus clade were found only, or nearly only, in marine and transition communities (Figure 4 and Figure 6). Interestingly, contigs from the clade of viruses infecting the marine chlorophyte *Micromonas* were found at higher abundances in transition compared to marine sites. Five contigs branched at the base of this clade; four of these were more abundant in freshwater and transition communities (0–3.7% of reads at these sites), and one was most abundant at a marine site (7.7% of reads at this site). Three contigs clustered within the genus *Chlorovirus*. Notably, no environmental sequences related to chloroviruses were retrieved from the IMG/VR database, although two short chlorovirus sequences were obtained by Short et al.’s study of Lake Ontario [23]. Two contigs from this clade were most abundant at the freshwater IDO site (32 and 58% of reads respectively), but moderately abundant at all other sites except the marine TRP where they were absent, while a third contig in the chlorovirus clade had its highest abundance (9.4% of virus reads) in the marine PAP site. No putative chlorovirus contigs were retrieved from the TRP site.

**Figure 7:**
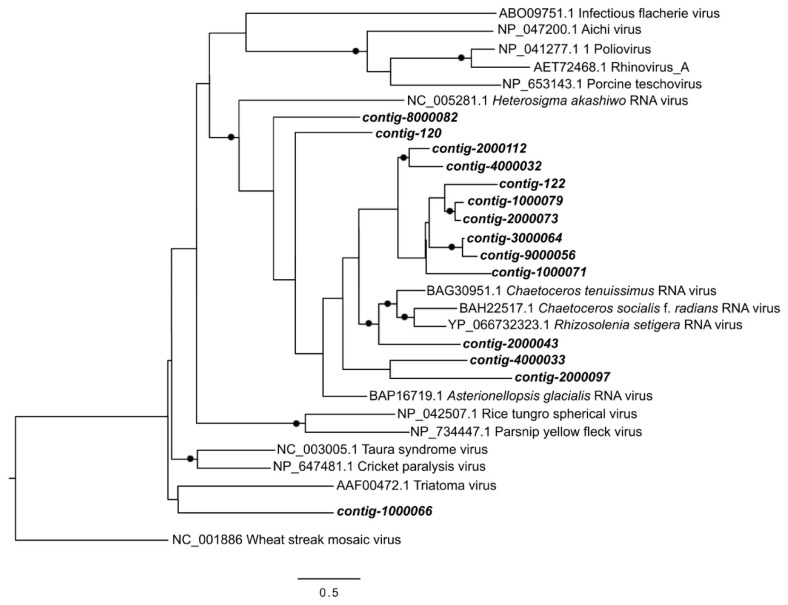
Maximum Likelihood tree showing the viral RNA-dependent RNA-polymerase (RdRp) contigs which contributed most to the dissimilarity between freshwater and brackish/saltwater samples in Similarity Percentage (SIMPER) analysis (>1% contribution). Closed circles show nodes with bootstrap values >50 (out of 100). Scale bar shows number of substitutions.

**Figure 8:**
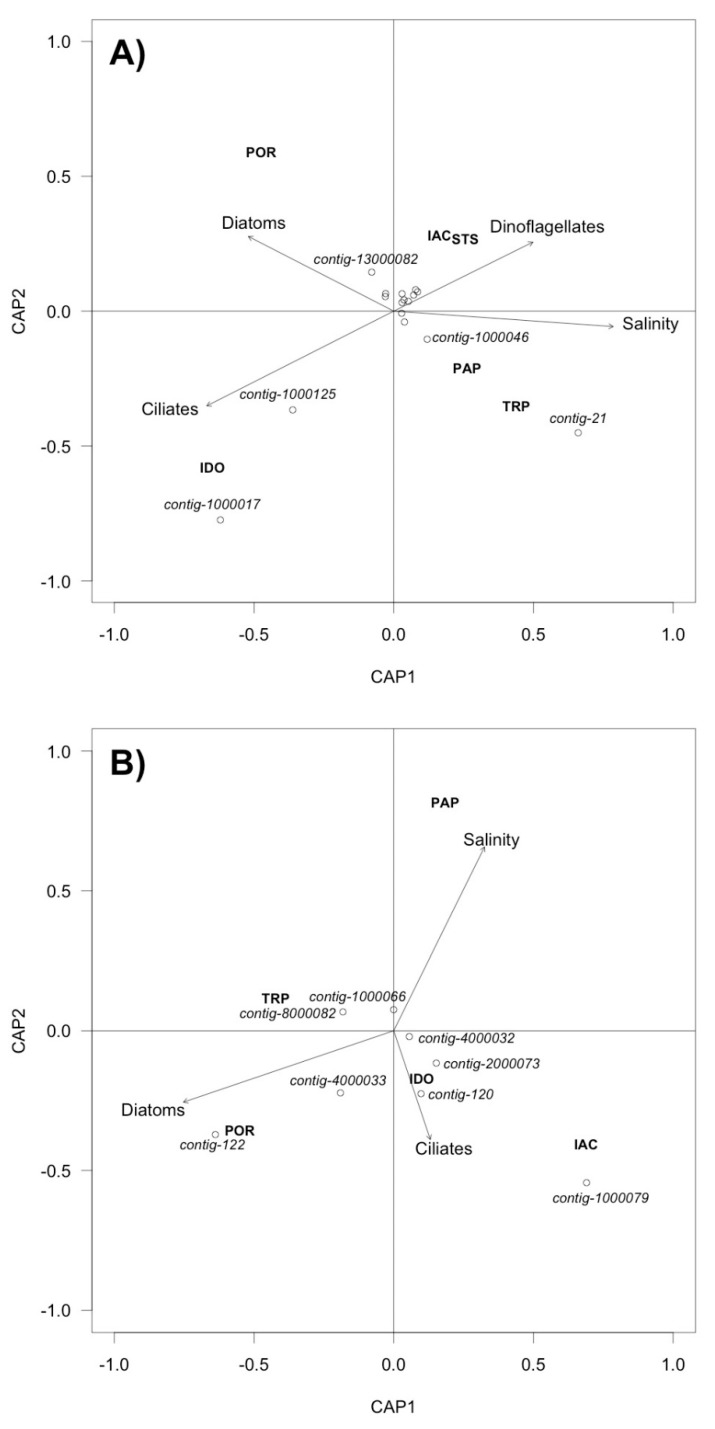
Triplots of distance-based redundancy analysis (db-RDA) using an unweighted UniFrac distance matrix for: (**A**) DNA virus contigs; (**B**) RNA virus contigs. Site scores are indicated by their three-letter code (see Table 2). Environmental variables are shown by arrows. Open circles show species scores for virus contigs. Some, but not all contigs are labelled.

The majority of RNA virus contigs (eleven contigs) were grouped within a clade of picornaviruses known to infect diatoms (Figure 7). Six of these were found only in marine sites, one was only in freshwater sites, three were found mostly in the brackish site IAC, and contig-122 was very abundant in both the freshwater POR (68% of virus reads) and marine TRP (25% of virus reads) sites (Figure 4). Two contigs were found at the base of this diatom virus clade, one was found only in a marine site, and one only in a freshwater site. Finally, one contig branched at the base of a clade of arthropod RNA viruses, and was found at moderate abundances (1–8%) in both marine and freshwater sites.

db-RDA analysis of DNA viruses produced a strong environmental axis separating fresh water rich in ciliates and diatoms, from salty water rich in dinoflagellates (Figure 8). For both DNA and RNA virus communities, the difference between diatom-dominated and ciliate-dominated freshwater sites was marked.

## 4. Discussion

Visual observations of turbidity showed that IDO was the sample site closest to the ETZ, which agrees with the coordinates found in previous studies [4,8]. High salinity at STS may have occurred because complex bottom topography at the mouth of Saguenay River, associated with the head of the Laurentian Channel, results in semidiurnal tidal upwelling of colder, saltier water [51], which would have been at its most intense at high tide.

There was no amplification of cDNA from STS, making it impossible to characterize the RNA virus community at this site. Humic substances have been identified as important PCR inhibitors [52], and may have occurred in higher concentrations around STS because of proximity to the Saguenay River outflow. STS was also characterized by the lowest relative abundance of diatoms of all the sites, possibly because of light limitation due to turbulent mixing in this area [5]. Since the majority of known marine picornaviruses infect diatoms, lower abundances of viral hosts may have combined with the presence of inhibitors to result in no amplification.

The diatom genus *Skeletonema* was a major component of the estuarine community, in agreement with the study of [4]. However, while they found that abundance of this genus increased with salinity, we found the reverse trend, with highest abundance (25% of RNA reads) at the freshwater site POR. The strain, or even species of *Skeletonema* likely differed between their study and ours. While it is generally considered to be a marine or brackish genus, some strains are known to tolerate salinities as low as 0, albeit with low growth [53]. Ciliates dominated the eukaryotic community at IDO, agreeing with a previous microscopic study of the ETZ that found that ciliates contributed up to 21% of eukaryote biovolume [2]. This earlier study identified ciliate genera *Strombidium* and *Strobilidium*, instead of the *Monodinium* and *Stokesia* which dominated in our IDO sample; they also found a high abundance of colourless chrysophytes, which did not appear in our samples. The primer set we used is known to have successfully recovered moderately high levels of chrysophyte sequences in an arctic lake [54]; thus, this difference is unlikely to be due to primer bias, and may reflect interannual variability in chrysophyte populations.

The eukaryotic community at STS clustered with marine sites in beta-diversity analysis, reflecting the fact that brackish environments are known to be dominated by marine, rather than freshwater, phytoplankton [5]. In contrast, for the DNA virus community, the two transition sites, STS and IAC clustered with each other, and contigs at these sites suggested a combination of influences, from both freshwater (contig-14000081, from a well-supported freshwater clade) and marine sources (putative *Micromonas* viruses). The higher abundance of contigs from the *Micromonas* virus clade, 2–3 times higher at transition compared to marine sites, did not follow abundances of *Micromonas*, which were higher at marine sites. One explanation may be that the *Micromonas* population in the transition sites was at a late stage of infection in which high numbers of virus particles co-occur with declining numbers of host cells.

The high number of RNA virus contigs unique to each site, and the low similarity between adjacent sites, agrees with a previous study of RNA viruses in a coastal environment which found even higher proportions of unique OTUs [55]. This difference between RNA and DNA viruses may reflect a higher degree of environmental specificity, with small differences between sites resulting in large differences in the RNA virus community; or it may be due to lower persistence of viral particles outside the host, which could limit dispersal between sites. Lower persistence would be consistent with speculation by [56] that RNA and DNA viruses of phytoplankton may represent r- and K-selected life strategies, respectively. However, the small number of viruses for which decay rates are known is insufficient to draw general conclusions [13].

In beta-diversity analysis of RNA viruses, IAC clustered with freshwater sites, even though it clustered with brackish and marine sites for eukaryotes. The most striking similarity between IAC and freshwater sites is abundance of the diatom genus *Skeletonema*, suggesting that this taxon may play a role in determining RNA virus distribution. Contig-4000033, whose relative abundance roughly tracks that of *Skeletonema* (Figure 4), may be a candidate to infect this diatom host.

Environmental sequencing studies have several intrinsic limitations, including differences in volumes filtered, potential presence of PCR inhibitors in natural samples, and the impossibility of reaching a saturated sampling curve for a diverse community. While these limitations mean that sequence data generated in this study can only be considered as being semi-quantitative, OTU relative abundance can still be meaningfully compared between sampling sites within the study.

Another limitation of our analysis is the paucity of reference sequences for viruses, and even of reliable environmental sequences, as demonstrated by the small number of hits in the IMG/VR database. While we can tentatively associate a given contig to a clade containing viruses with known hosts, it remains probable that some of these identifications will be invalidated as more reference sequences are added in the future. Compounding this problem, there is evidence that the AVS-1 and AVS-2 primers used in this study have a bias toward prasinoviruses [57]. The taxonomic coverage of Chlvd primers remains poorly known; they were specifically designed for the chlorovirus genus [23], but in our study they also amplified non-chlorovirus taxa, such as contigs 1000046 and 10000103. Our results confirm that different primer sets amplify different virus populations (Supplementary Figure S1). With these caveats, our identification of a prasinovirus clade (Figure 6) is strengthened by the observation that the eight contigs that map to it have a distribution congruent with the distribution of Mamiellophyceae, being absent or nearly absent from the freshwater sites (Figure 4).

Similar caveats apply to identifying three contigs, 1000125, 1000017, 3000065, with a clade of chloroviruses infecting the symbionts of ciliates (Figure 5). The total absence of a potential *Chlorella*-symbiont host in the eukaryote 18S sequences would argue against this identification. However, *Chlorella* sequences have been detected upstream in Lake Ontario in previous years [23], so their presence cannot be ruled out. It is also notable that db-RDA associated two of these contigs with high ciliate abundance, owing to their high concentration at the site IDO (Figure 8).

## 5. Conclusions

This first viral study of the estuarine transition zone of the St. Lawrence hydrographic system found phycodnaviruses and picorna-like viruses in freshwater, transition, and estuarine locations. Although some OTUs from this study were related to viruses in the genera *Chlorovirus* and *Prasinovirus*, most of the DNA OTUs and all of the RNA OTUs appeared to be divergent from classified viruses. In general, both the eukaryotic and viral community composition showed a pronounced difference between freshwater sites and the rest of the transect; however, beta-diversity and the abundance of specific contigs did not always follow salinity trends, indicating that other processes, such as viral life cycle or horizontal advection, may play a role producing the observed distributions. The sequences and associated ecological data from our study represent a substantial contribution to impoverished viral sequence databases.

## Figures and Tables

**Table 1:** Primers used in this study.

*Primers targeting RNA-dependent RNA-polymerase (Picornavirales):*			
**Primer**	**Sequence**	**Hybridization Temperature (°C)**	**Reference**
Mpl.sc2F	ITWGCIGGIGATTWCA	43.3	[21]
Mpl.sc2R	CKYTTCARRAAWTCAGCATC	43.3	[21]
RdRp1	GGRGAYTACASCIRWTTTGAT	50	[21]
RdRp2	MACCCAACKMCKCTTSARRAA	50	[21]
*Primers targeting DNA-dependent DNA polymerase (Phycodnaviridae):*			
**Primer**	**Sequence**	**Hybridization Temperature (°C)**	**Reference**
AVS1	GARGGIGCIACIGTIYTIGAYGC	44.9	[22]
AVS2	GCIGCRTAICKYTTYTTISWRTA	44.9	[22]
ChlvdF	CCWATCGCAGCWCTMGATTTTG	52	[23]
ChlvdR	ATCTCVCCBGCVARCCACTT	52	[23]

**Table 2:** Site salinity (as S_P_) and number of viral sequences (Seqs.) and contigs (treated as Operational Taxonomic Units (OTUs)) from stations along a longitudinal transect of the St. Lawrence Estuary. The percentage of contigs unique to a given site is also given.

		DNA Viruses			RNA Viruses		
Station	Salinity	Seqs.	Contigs	Unique Contigs (%)	Seqs.	Contigs	Unique Contigs (%)
Portneuf (POR)	0.11	369,201	316	4.7	70,103	29	34
Île d’Orléans (IDO)	0.13	12,264	99	0	28,605	17	18
Isle-aux-Coudres (IAC)	23.17	86,707	252	0.4	705,577	35	26
St-Siméon (STS)	28.77	1,371,765	387	3.3	NA	NA	NA
Trois-Pistoles (TRP)	29.37	2,397,455	381	3.8	4368	34	26
Pointe-au-Père (PAP)	29.57	3,494,306	392	2.8	15,526	37	36

**Table 3:** Similarity percentage (SIMPER) analysis listing the top 12 eukaryotic OTUs which contributed to the dissimilarity between freshwater (POR, IDO) and transition and marine sites (IAC, STS, TRP, PAP; full names of sites given in Table 2) for DNA- and RNA-amplified samples. (Cum. = Cumulative).

	Contribution			Contribution	
DNA	%	Cum.	RNA	%	Cum.
Ciliate—Choreotrichida	11.3	11.3	Diatom—*Skeletonema*	12.9	12.9
Diatom—*Skeletonema*	10.4	21.6	Chlorophyte—*Ostreococcus*	8.3	21.2
Diatom—Thalassiosirales	7.3	28.9	Cryptophyte—*Cryptomonas*	8.3	29.4
Dinoflagellate—*Heterocapsa rotundata*	6.2	35.2	Ciliate—*Stokesia*	6.4	35.8
Marine Alveolate—Unclassified	5.8	40.1	Cryptophyte—*Plagioselmis/Teleaulax*	6.0	41.8
Dinoflagellate—Gymnodiniales	5.2	46.1	Dinoflagellate—*Heterocapsa rotundata*	5.5	47.3
Marine Alveolate—Guillou II.1	4.6	50.7	Cryptophyte—*Teleaulax gracilis*	5.4	52.7
Chlorophyte—*Ostreococcus*	4.5	55.2	Chlorophyte—Mamiellophyceae	3.9	60.5
Cryptophyte—*Cryptomonas*	2.8	61.7	Diatom—Thalassiosirales	2.4	63.0
Picozoa—NW617.02	2.2	63.9	Diatom—*Thalassiosira*	2.4	65.3
Ciliate—Oligotrichida	2.2	66.0	Picozoa—NW617.02	2.4	68

## Supplementary Materials

The following are available online at http://www.mdpi.com/1999-4915/10/12/672/s1, Figure S1: Relative amplification of viral contigs by different primer sets for (A) DNA viruses (ChlVd and AVS primers); (B) RNA viruses (Mpl.Sc2 and RdRp primers).
