# Supplementary material for: Communities of Phytoplankton Viruses across the Transition Zone of the St. Lawrence Estuary
Source: Viruses. 2018 Nov 27;10(12):672. doi: 10.3390/v10120672 (PMC6316209; doi:10.3390/v10120672)
Supplement: Supplementary file 1 [file viruses-10-00672-s001.pdf]

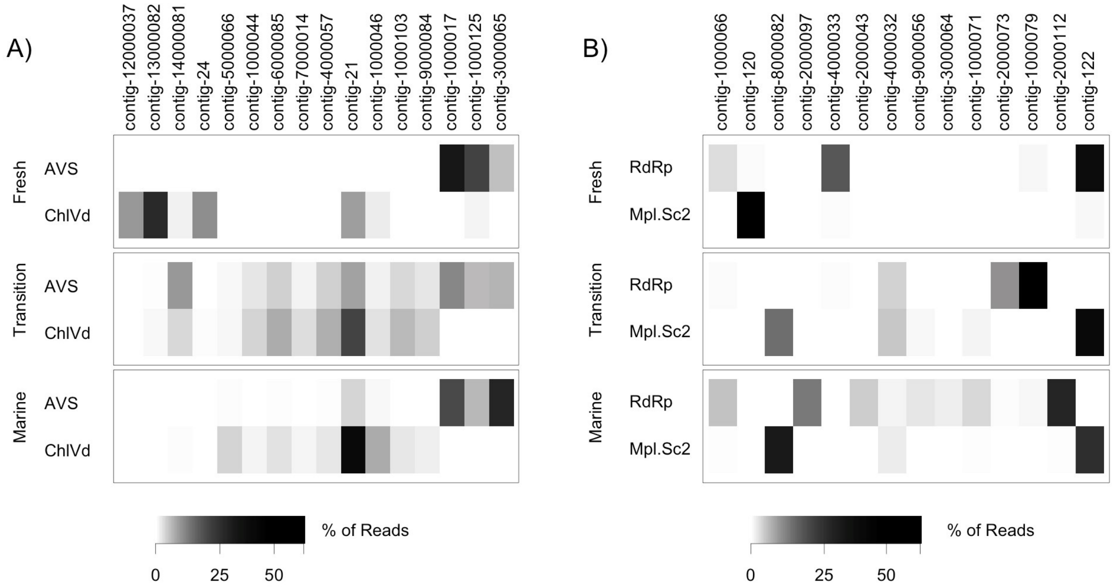

Figure S1. Relative amplification of viral contigs in marine, transition zone and freshwater sites, by different primer sets for (**A)** DNA viruses (ChlVd and AVS primers); (**B**) RNA viruses (Mpl.Sc2 and RdRp primers).
